# Salter‐Harris type II fracture of the distal femur in a newborn: Acute anatomic imaging alterations after labor dystocia

**DOI:** 10.1002/ccr3.2149

**Published:** 2019-05-16

**Authors:** Nicolaos Laliotis, Stylianos Kapetanakis, Grigorios Gkasdaris, Panagiotis Konstantinidis, Ritsa Karoutsou, Danae Chourmouzi, Lamprini Giannakopoulou

**Affiliations:** ^1^ European Interbalkan Medical Center Thessaloniki Greece; ^2^ Radiology Department European Interbalkan Medical Center Thessaloniki Greece

**Keywords:** anatomy, femur, fracture, newborn, Salter‐Harris, type II

## Abstract

A rare clinical presence of Salter‐Harris type II fracture of the distal femur in a newborn. The crucial role of imaging in depicting urgent anatomical alterations.

## 1 CASE DESCRIPTION

**Figure 1:**
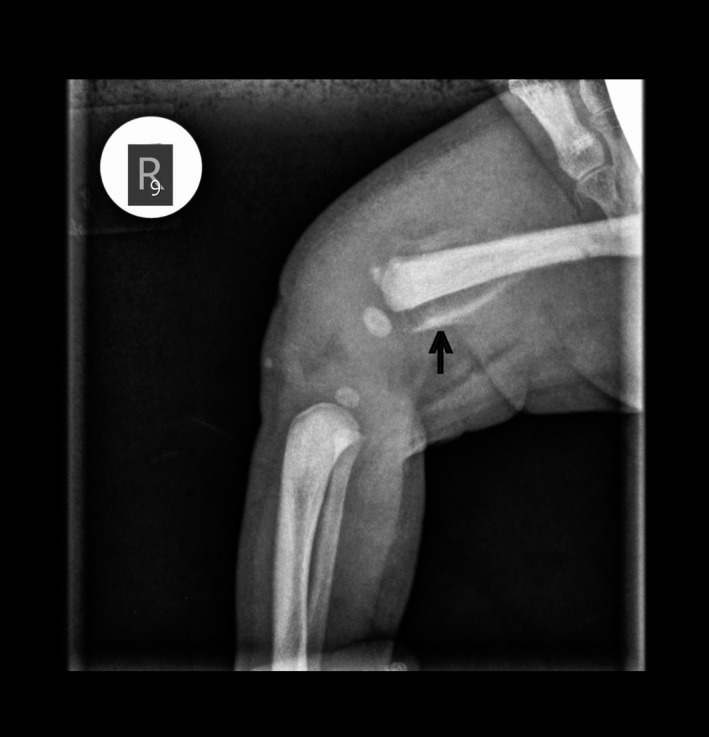
X‐ray of the right femur. The black arrow indicates the fracture and the detached osseous part

**Figure 2:**
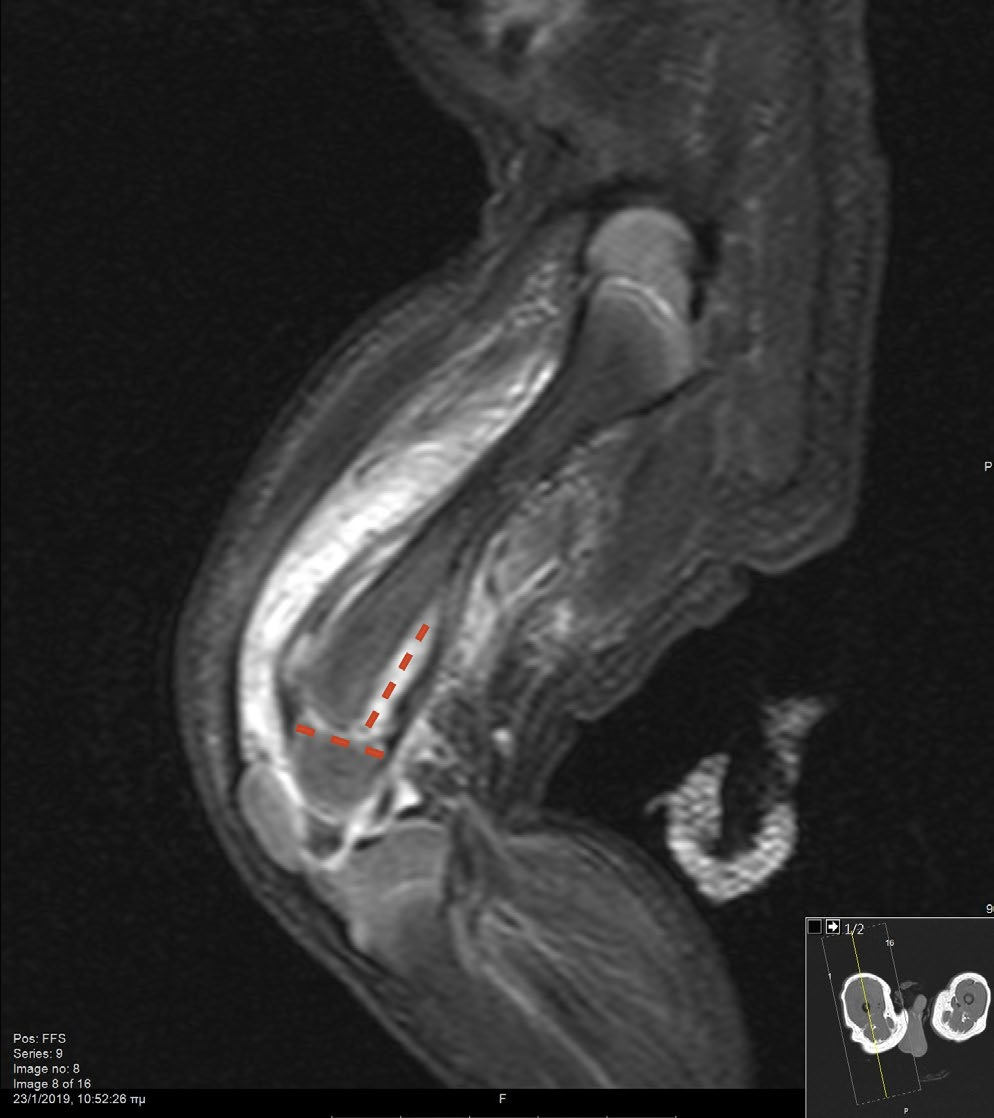
MRI of the right femur depicting the presence of hematoma and disruption of periosteum, setting the diagnosis of Salter‐Harris type II fracture. The red lines indicate the type of the fracture

A baby boy was delivered with breech birth after difficult labor. Before birth, the imaging examinations of pregnancy were normal. Immediately after birth, within the first 24 hours, the newborn presented difficulty in movement of the right leg. Edema and pain in the distal third of the femur were observed. New imaging studies were performed. The ultrasound scan was normal. The X‐rays set the suspicion of a fracture (Figure 1). In the next day, the MRI scan confirmed the presence of a Salter‐Harris type 2 fracture in the distal third of the right femur (Figure 2). The newborn was treated with a cast and immobilization. At the one month follow‐up, the clinical and radiological examinations have indicated a successful outcome.

Distal femoral physis fractures with displacement are rare injuries seen mainly in adolescents. Premature physeal closure, growth arrest, and bone deformity are possible complications.^1^ As regards to the surgical management, the epiphysis can be fixed with two screws introduced above the growth plate or with the use of Kirschner wires inserted through epiphysis.^2^, ^3^ After discussion with the parents, we jointly chose the conservative treatment in this specific case. Our case is unique and very rare, due to the fact that the fracture is presented immediately after birth in the distal part of the femur of a newborn and due to the choice of a successful alternative management. Imaging studies, and especially MRI, have a crucial role. The management in the very first days of life is very challenging.

## CONFLICT OF INTEREST

None declared.

## AUTHOR CONTRIBUTION

NL: identified the case. SK: planned the manuscript structure. GG: prepared the manuscript. PK: reviewed the literature. RK: assessed the pediatric aspect of the case. DC: assessed the imaging findings. LG: assessed the radiologic aspect of the case.
